# Histology, immunohistochemistry, and *in situ* hybridization reveal overlooked Ebola virus target tissues in the Ebola virus disease guinea pig model

**DOI:** 10.1038/s41598-018-19638-x

**Published:** 2018-01-19

**Authors:** Timothy K. Cooper, Louis Huzella, Joshua C. Johnson, Oscar Rojas, Sri Yellayi, Mei G. Sun, Sina Bavari, Amanda Bonilla, Randy Hart, Peter B. Jahrling, Jens H. Kuhn, Xiankun Zeng

**Affiliations:** 1 Integrated Research Facility at Fort Detrick, National Institute of Allergy and Infectious Diseases, National Institutes of Health, Fort Detrick, Frederick, Maryland USA; 2 United States Army Medical Research Institute of Infectious Diseases, Fort Detrick, Frederick, Maryland USA; 3 Present Address: Path-2-Gene, LLC, Harrisburg, PA USA

**Keywords:** Viral infection, Infection

## Abstract

Survivors of Ebola virus infection may become subclinically infected, but whether animal models recapitulate this complication is unclear. Using histology in combination with immunohistochemistry and *in situ* hybridization in a retrospective review of a guinea pig confirmation-of-virulence study, we demonstrate for the first time Ebola virus infection in hepatic oval cells, the endocardium and stroma of the atrioventricular valves and chordae tendinae, satellite cells of peripheral ganglia, neurofibroblasts and Schwann cells of peripheral nerves and ganglia, smooth muscle cells of the uterine myometrium and vaginal wall, acini of the parotid salivary glands, thyroid follicular cells, adrenal medullary cells, pancreatic islet cells, endometrial glandular and surface epithelium, and the epithelium of the vagina, penis and, prepuce. These findings indicate that standard animal models for Ebola virus disease are not as well-described as previously thought and may serve as a stepping stone for future identification of potential sites of virus persistence.

## Introduction

Ebola virus disease (EVD) is a severe and frequently lethal affliction of humans caused by infection with any of three members of the mononegavirus family *Filoviridae*: Bundibugyo virus (BDBV), Ebola virus (EBOV), and Sudan virus (SUDV). A fourth virus, Taï Forest virus (TAFV), has thus far caused only a single reported human infection, which was nonlethal^1^. EVD is an exotic disease with case numbers rarely surpassing the lower hundreds^1^; however, from 2013–2016, EBOV caused an EVD outbreak in Western Africa encompassing 28,616 infections and 11,310 deaths in Guinea, Liberia, and Sierra Leone^2^. Long term sequelae in individual survivors of acute EVD and the similar Marburg virus disease (MVD) and filovirus persistence followed by disease relapse or sexual transmission had been reported before this outbreak^3–8^. However, observations during and following the Western African EVD outbreak suggest that sequelae and filovirus persistence may be common events^9^. Reported sequelae include arthralgia, cardiac valvulopathy, parotid gland inflammation, peripheral paresthesia or dysesthesia, and gastrointestinal motility disorders^10–14^. Semen may contain detectable EBOV RNA for more than 500 days following recovery, and EBOV RNA has been detected in breast milk of a subclinically infected mother^15,16^. Replicating EBOV has been isolated from the cerebrospinal fluid of an EVD survivor suffering a disease relapse and from the aqueous humor of the eye of another survivor^17,18^. Sexual EBOV transmission from EVD survivors to partners months after infection also has been documented^19,20^.

Only 26 EVD outbreaks (16 of them due to EBOV infection) have been recorded since the initial discovery of EVD-causing viruses in 1976, and excluding the Western African outbreak, no EVD outbreak has encompassed more than 500 cases^1^. Because the natural host reservoirs of EVD-causing filoviruses remain to be identified, the periodic introductions of these viruses into the human population are unpredictable and unpreventable. In conjunction with the high case-fatality rate of EVD^1^, performance of meaningful clinical studies is challenging. Therefore, characterization of EVD pathogenesis and primary development and evaluation of medical countermeasures largely relies on experimental infections of animal models. Predominantly, these models include nonhuman primates (crab-eating, aka cynomolgus, macaques [*Macaca fascicularis*] and rhesus monkeys [*Macaca mulatta*]), and, after virus adaptation, rodents (laboratory mice, guinea pigs [*Cavia porcellus*], and Syrian hamsters [*Mesocricetus auratus*])^21,22^. Recently, domestic ferrets (*Mustela putorius furo*) have been identified as novel EVD animal models not requiring virus adaptation^23–25^. However, with the exception of one study demonstrating EBOV persistence in apparently healthy rhesus monkey survivors of EBOV-induced disease^26^, development of sequelae and/or viral persistence, and demonstration of sexual transmission, remains to be recapitulated in animal models.

Based on *in vitro* results of studies of EBOV persistence in cell culture^27^, we speculate that EBOV may establish *in vivo* persistent infections in any entry-permissive cell type, rather than only in immunoprivileged sites such as brain, eyes, and testes. Before this speculation can be tested, validating the reported tissue and cell tropism of EBOV in animal models is imperative. We chose the guinea pig model, as the model is considered to be more stringent than the laboratory mouse model and less ethically, financially, and logistically burdensome than nonhuman primates models^21,22^. The guinea pig model is considered well-characterized and is actively used for transmission and medical countermeasure evaluation studies. EBOV infection has been previously demonstrated in a wide variety of guinea pig tissues and cell types, including cells of the monocyte or macrophage system, hepatocytes, fibroblastic reticular cells, interstitial fibroblasts, adrenal cortical cells, ovarian thecal cells, endothelial cells, endometrial stromal cells, and cells of the urothelium^28–39^. In a retrospective histologic review of tissues from EBOV-infected guinea pigs, in combination with immunohistochemistry and *in situ* hybridization, we demonstrate that EBOV infection of several tissues and numerous cell types has been overlooked.

## Results

All 30 guinea pigs intraperitoneally infected with 1 of 3 EBOV doses (n = 10/group) developed illness. Seven guinea pigs (3 in 10-PFU group, 2 in 100-PFU group, and 2 in the 1,000-PFU group) succumbed to EVD and were not necropsied due to autolysis. The remaining 23 guinea pigs (7 in the 10-PFU group, 8 in the 100-PFU group, and 8 in the 1,000-PFU group) were necropsied, and specific tissues were collected for histology (see Supplemental Table 1 for animal-by-animal breakdown of sex, inoculation dose, day of euthanasia, and relevant pathology findings). All 23 guinea pigs examined histologically had extensive hepatocellular and lymphoid necrosis consistent with previous descriptions of guinea pig-adapted Ebola virus infection in this model^30,31^. IHC and ISH staining confirmed hepatocytes, monocytes and macrophages as major targets of EBOV infection.

**Table 1:** Experimental endpoint criteria.

Score	Criteria*
0	No evidence of pain or distress. Normal activity.
1	Mild or anticipated pain or distress. Examples of clinical signs: hiding or decreased activity, soft tissue swelling or discomfort without other signs, abnormal or hunched posture, or unthrifty, ungroomed or scruffy appearance, but active.
2	Moderate pain or distress. Examples of clinical signs: rapid or shallow respiratory rate, head tilt, paresis (limb weakness, difficulty in ambulation), abnormal nesting behavior, abnormal or hunched posture, or unthrifty, ungroomed or scruffy appearance with reduced activity, but responsive when stimulated.
3	Severe pain or distress. Examples of clinical signs: pale skin or mucous membranes, unable or unwilling to move or comatose (moribund) when stimulated, agonal breathing, paralysis, head tilt with circling or rolling, persistent scratching, abnormal or hunched posture or unthrifty, or ungroomed or scruffy appearance.

^*^Scoring is based on the presence of one or more clinical signs within the example list. Guinea pigs were considered moribund and were euthanized after reaching a score of 3.

### Liver

**Figure 1:**
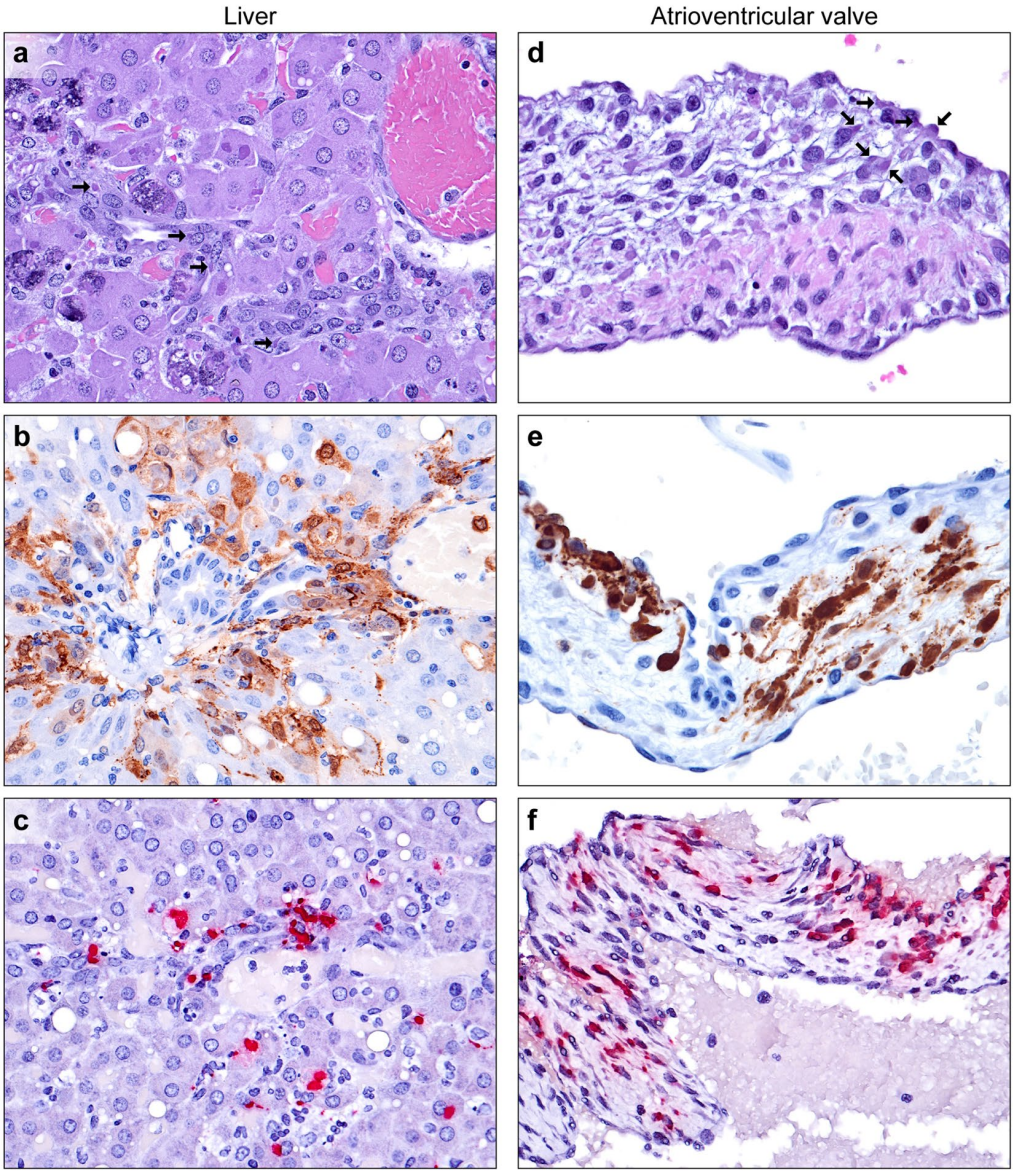
EBOV infection of the liver and heart. (**a**) Oval cell infection of the liver. Parallel streams of basophilic oval cells form duct-like structures adjacent to a portal triad, with frequent large eosinophilic toamphiphilic intracytoplasmic inclusion bodies (arrows, H&E). (**b**) Proliferating oval cells, hepatocytes, Kupffer cells, and intravascular monocytes (EBOV VP40 IHC: DAB chromogen and hematoxylin). (**c**) Oval cells, hepatocytes, and Kupffer cells (EBOV NP ISH: fast red chromogen and hematoxylin). (**d**) Valvular endocarditis of tricuspid valve cusp. Degeneration and necrosis of surface (atrialis) endocardial cells and stromal cells of the spongiosa, with infiltrating macrophages and frequent large eosinophilic to amphiphilic intracytoplasmic inclusion bodies (arrows, H&E). (**e**) Endocardial cells, stromal cells, and macrophages (EBOV VP40 IHC: DAB chromogen and hematoxylin). (**f**) Endocardial cells, stromal cells, and macrophages (EBOV NP ISH: fast red chromogen and hematoxylin).

The majority of findings were consistent with previous reports^30,31^, including widespread hepatocyte and Kupffer cell infection and necrosis, with variable dystrophic mineralization. In contrast to the rhesus monkey model of EVD, viral intracytoplasmic inclusion bodies (ICIB) were large and numerous. Livers were collected from 6/7 guinea pigs in the 10-PFU, 8/8 in the 100-PFU, and 8/8 in the 1,000-PFU group (Supplemental Table 1). In 7 of 22 livers examined (samples from 1 guinea pig were not available), multifocal minimal- to-moderate oval cell hyperplasia originated in the biliary ductules and extended into lobules. Small basophilic cells with a high nucleus:cytoplasm ratio formed parallel streams and duct-like structures, consistent with International Harmonization of Nomenclature and Diagnostic criteria (INHAND)^40^. Viral ICIB were frequently observed within oval cells (Fig. 1a) with IHC and ISH positive signals (Fig. 1b,c). Oval cells were present in animals from all three dosage groups, with the occurrence of such cells loosely correlating with the infectious inoculum: 1 of 6 guinea pigs in the 10-PFU group; 1 of 8 in the 100-PFU group; and 5 of 8 in the 1000-PFU group. Oval cells were only present in animals necropsied at d 8 or 9 post-inoculation. No viral infection of biliary ductules was present as judged by histology, IHC, or ISH.

### Heart

Cross-sections through one or both ventricles were collected from 22 of 23 guinea pigs (samples from 1 guinea pig were not available), with variable inclusion of atrioventricular valves and associated chordae tendinae; semilunar valves were not sampled. In all 3 examined tricuspid valves (one each from the 10-, 100-, and 1,000-PFU groups) and both mitral valves (one each from the 100- and 1000-PFU groups), viral ICIB were present in the stromal spindled fibroblasts of the spongiosa and surface endocardium of atrialis of the atrioventricular valves (Fig. 1d). Sometimes viral ICIBs were associated with infiltrating macrophages and apoptotic and necrotic cellular debris (Fig. 1d). EBOV infection of the fibroblasts was confirmed by IHC and ISH (Fig. 1e,f). Viral ICIBs were also present in the stromal cells of the chordae tendinae of the tricuspid valve in 8 of 11 examined guinea pigs and of the chordae tendinae of the mitral valve of 16 of 17 examined guinea pigs. In three of 22 guinea pigs, very rare individual cardiomyocytes were noted that contained ICIB, with confirmation by IHC.

### Nerves and ganglia

**Figure 2:**
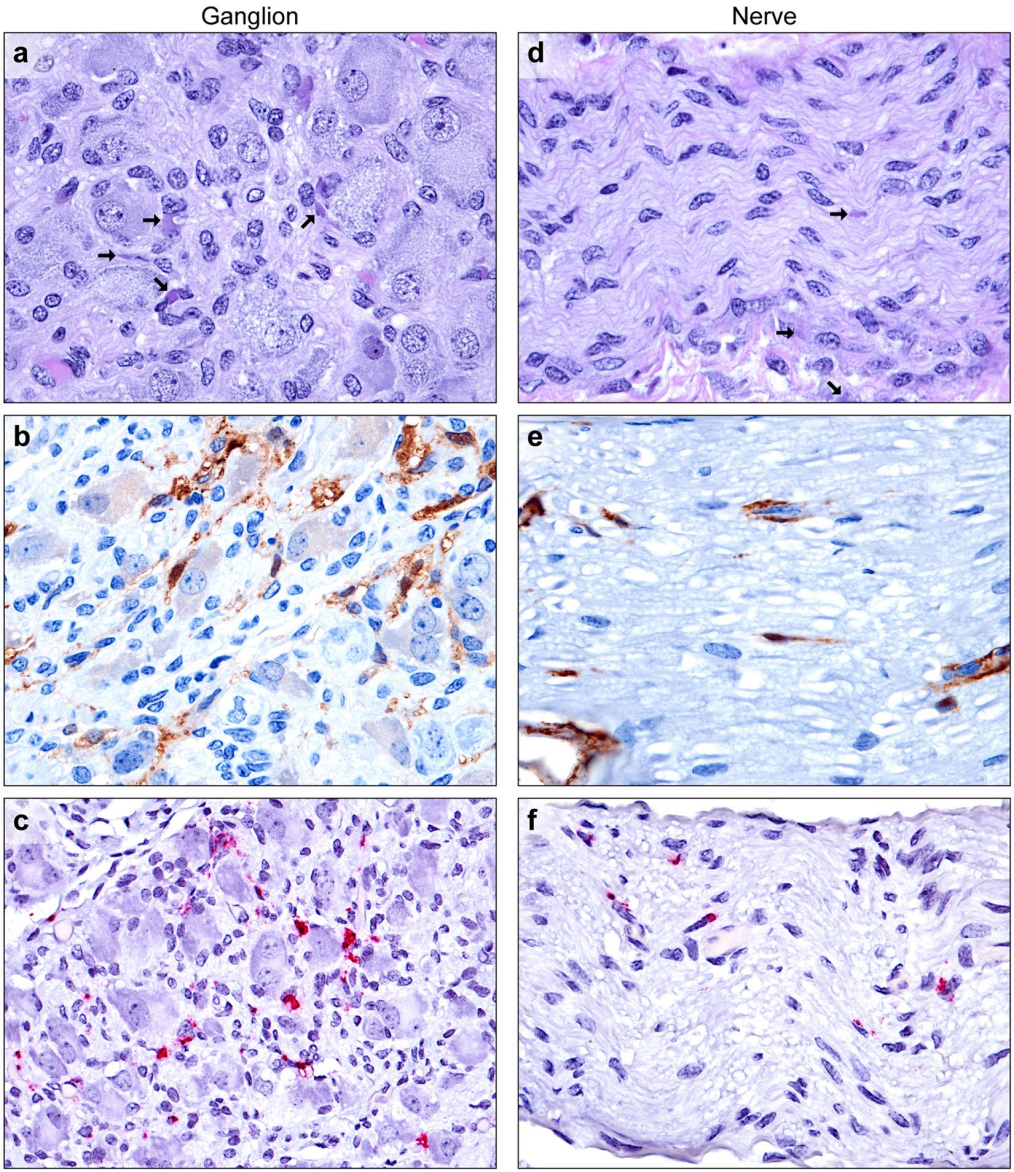
EBOV infection of paradrenal ganglia and peripheral medium myelinated nerves. (**a**) Low numbers of macrophages infiltrate between ganglion cells in the paradrenal ganglion. Viral intracytoplasmic inclusion bodies (arrows) are present within satellite cells, neurofibroblasts, Schwann cells, and macrophages (H&E). (**b**) Satellite cells, neurofibroblasts, Schwann cells, and macrophages, and intravascular plasma (EBOV VP40 IHC: DAB chromogen and hematoxylin). (**c**) Satellite cells, neurofibroblasts, Schwann cells, and macrophages in the ganglion (EBOV NP ISH: fast red chromogen and hematoxylin). (**d**) Viral intracytoplasmic inclusion bodies (arrows) are present within neurofibroblasts and Schwann cells within a nerve, with low numbers of perivascular infiltrating macrophages (bottom) (H&E). (**e**) Rare infected Schwann cells and neurofibroblasts within a myelinated nerve (EBOV VP40 IHC: DAB chromogen and hematoxylin). (**f**) Low numbers of Schwann cells, neurofibroblasts, and infiltrating macrophages within a myelinated nerve (EBOV NP ISH: fast red chromogen and hematoxylin).

From samples available from 7 guinea pigs, the paradrenal or mesenteric ganglia and nerves were collected with adrenals or mesenteric lymph nodes, respectively. Multifocal mild to rarely moderate histiocytic inflammation (ganglioneuritis) was noted in 5 of 7 animals, with mild ganglion cell degeneration or loss and satellite cell hyperplasia (nodules of Nageotte). Viral ICIB were present within infiltrating macrophages, and occasional satellite cells, neurofibroblasts, and Schwann cells (Fig. 2a), with EBOV-positive labeling by IHC and ISH (Fig. 2b,c). No evidence of neuronal infection was observed in any sample. Multiple myelinated medium and large mesenteric nerves and other peripheral nerves were characterized by rare ICIB within neurofibroblasts, Schwann cells, and infiltrating macrophages (Fig. 2d). Infected cells, endothelial cells, and intravascular plasma were labeled by IHC and ISH (Fig. 2e,f). In a single guinea pig, multiple digestion chambers consisting of segmental expansion of the axon sheath by lipid laden macrophages were present in a single large axillary/brachial nerve in the absence of ICIB. Unfortunately, the nerve was lost on re-sectioning.

### Genital tract

**Figure 3:**
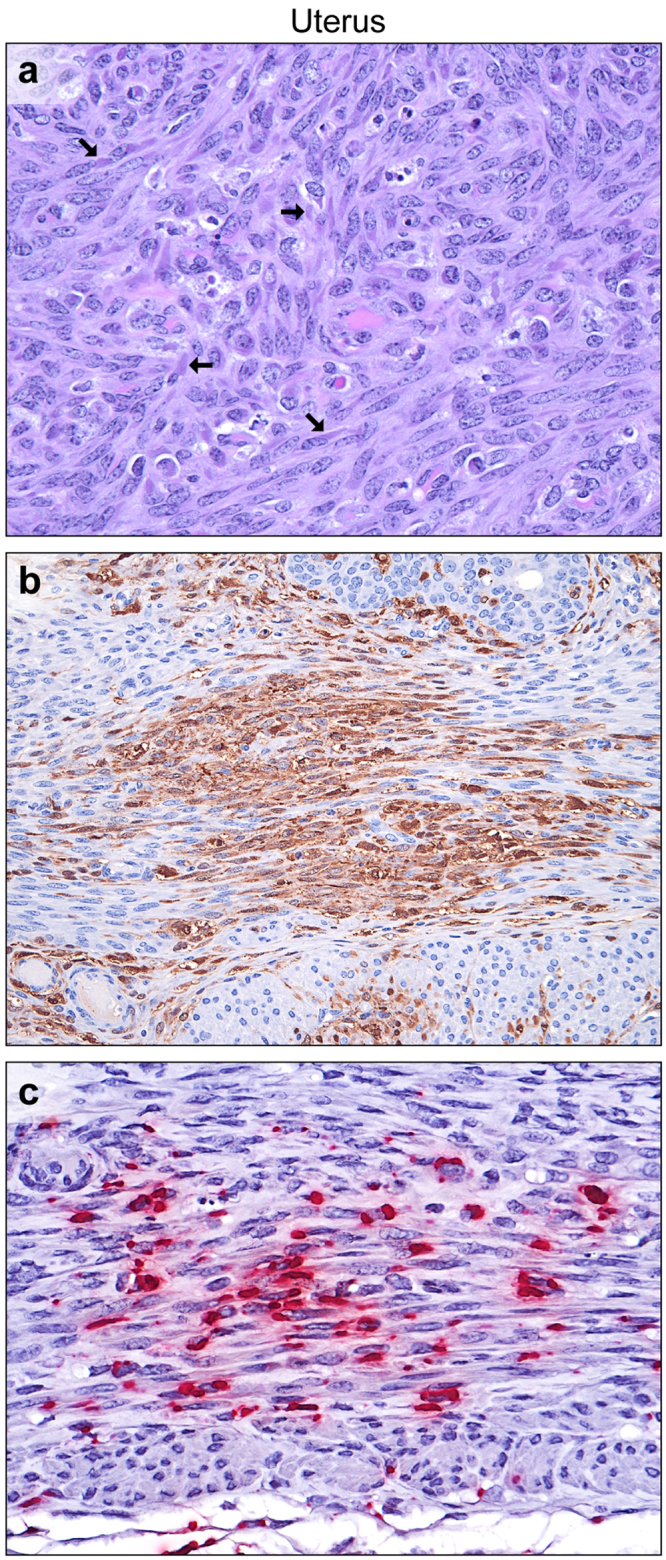
EBOV infection of the uterus. (**a**) Numerous large eosinophilic to amphiphilic intracytoplasmic inclusion bodies (arrows) within smooth myocytes, with scattered apoptosis and necrosis (H&E). (**b**) Smooth myocytes and endometrial stromal cells (EBOV VP40 IHC: DAB chromogen and hematoxylin). (**c**) Myometrial smooth muscle cells (EBOV NP ISH: fast red chromogen and hematoxylin).

**Figure 4:**
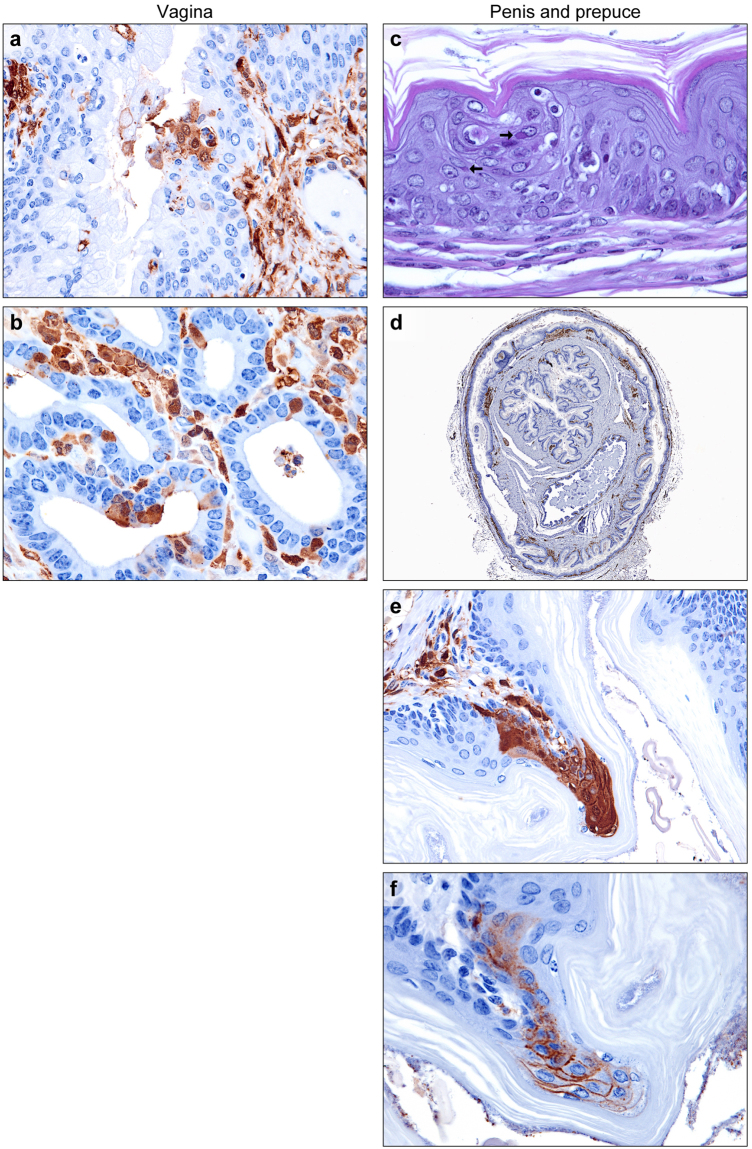
EBOV infection of the vagina, penis, and prepuce. (**a**) Rare small clusters of infected metestrus epithelial cells in the vagina (EBOV VP40 IHC: DAB chromogen and hematoxylin). (**b**) Endometrial gland epithelial cells, as well as stromal cells and macrophages, of the uterine body (EBOV VP40 IHC: DAB chromogen and hematoxylin). (**c**–**f**) Penis and prepuce. (**c**) Scattered small clusters of keratinocytes within the stratified squamous cornifying epithelium with large viral intracytoplasmic inclusions (arrows). (**d**) In addition to stromal fibroblasts and macrophages, clusters of epithelial cells covering the penis and prepuce (EBOV VP40 IHC: DAB chromogen and hematoxylin). (**e**) Higher magnification of subfigure. (**d**,**f**) Clusters of infected epithelial cells. (EBOV GP IHC: DAB chromogen and hematoxylin).

Of the 12 EBOV-infected female guinea pigs for which slides were available, uterine bodies were collected from 3 animals, uterine cervices and vaginas from 3 animals, and vaginas from 2 animals. In all specimens examined except one vagina, rare-to-frequent viral ICIB were present within smooth muscle cells, with scant apoptosis, necrosis, or histiocytic inflammation (Fig. 3a–c). In two sections of uterus and two sections of vagina, immunolabeling of rare small clusters of surface and glandular epithelial cells was positive (Fig. 4a,b). Consistent with previous reports, endometrial stromal cells and interstitial fibroblasts, macrophages and endothelial cells contained ICIB and stained positively for EBOV by IHC and ISH^30,31^.

The penis and prepuce was collected from a single male guinea pig (post-inoculation day 9, 100-PFU group). Several small clusters of spinous layer epithelial cells in the prepuce exhibited cytoplasmic vacuolation and contained viral ICIB (Fig. 4c). Scattered apoptosis and necrosis of adjacent cells were noted. Discrete vertical columns of EBOV-positive cells (IHC) were present in the non-haired skin of both the penis and prepuce (Fig. 4d–f).

### Salivary glands

**Figure 5:**
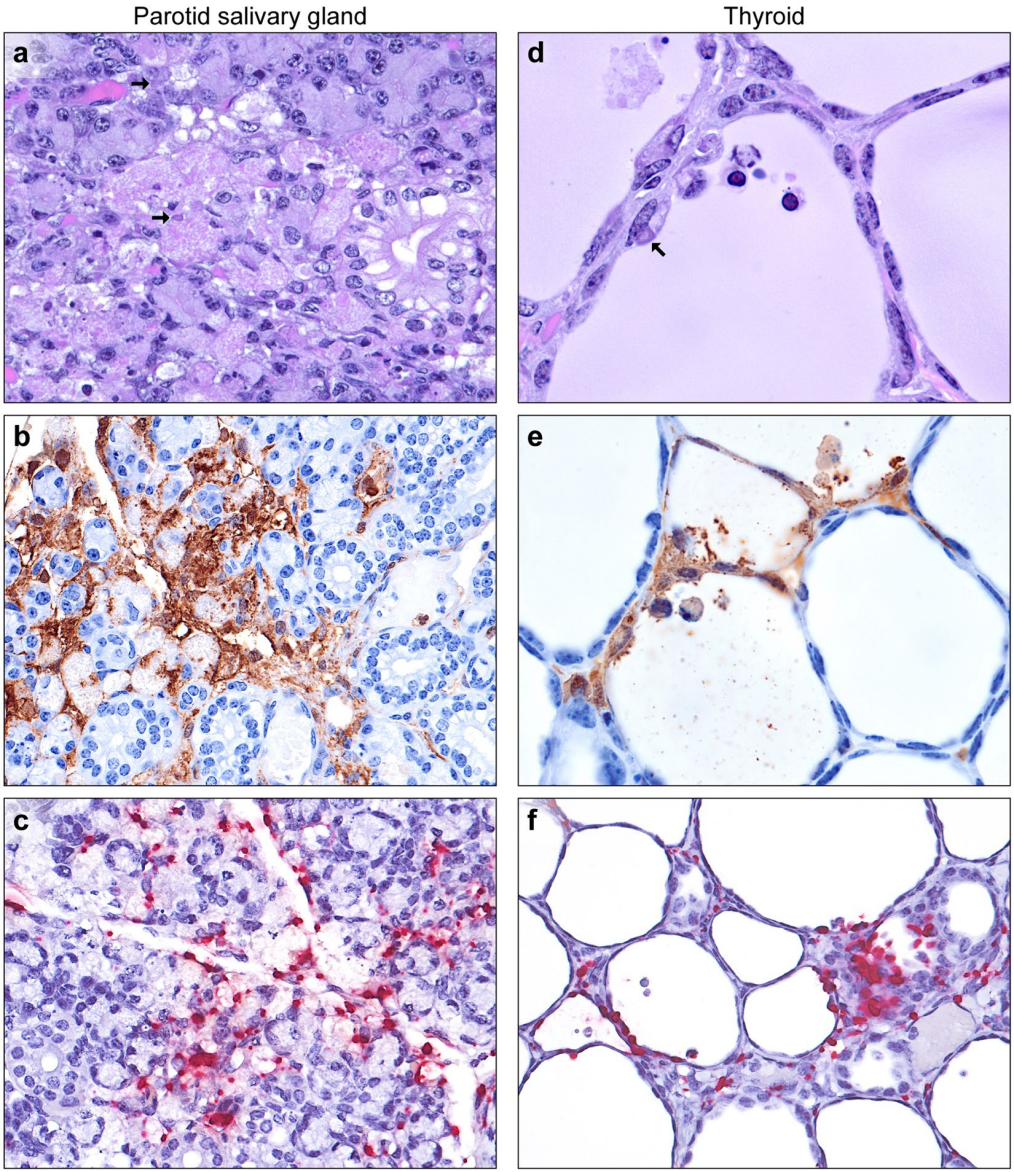
EBOV infection of the salivary glands and thyroid. (**a**) Necrotizing adenitis of parotid salivary gland. Focal acinar cell necrosis with rare large eosinophilic to amphiphilic intracytoplasmic inclusion bodies (arrows, H&E). (**b**) Infiltrating macrophages, interacinar fibroblasts, and viable and degenerate acinar cells (EBOV VP40 IHC: DAB chromogen and hematoxylin). (**c**) Infected acinar cells, macrophages and fibroblasts (EBOV NP ISH: fast red chromogen and hematoxylin). (**d**) Degeneration, sloughing, and necrosis of follicular epithelium, with rare large eosinophilic to amphiphilic intracytoplasmic inclusion bodies (arrows) in follicular cells and infiltrating macrophages (H&E). (**e**) Infected follicular cells, intra-follicular and interstitial macrophages, and interstitial stromal cells (EBOV VP40 IHC: DAB chromogen and hematoxylin). (**f**) Infected follicular epithelium and interstitial cells (EBOV NP ISH: fast red chromogen and hematoxylin).

Salivary glands were collected incidentally with the mandibular lymph nodes from some animals. In most salivary glands, very low numbers of macrophages and fibroblasts containing ICIB were present in the interstitium surrounding the acini and ducts. In 8 of 10 parotid glands examined, multifocal mild-to-moderate acinar cell necrosis with histiocytic inflammation was observed (Fig. 5a). Immunostaining of 3 glands showed abundant EBOV antigen localized to these necrotic foci and in viable and degenerate acinar cells (Fig. 5b). ISH results confirmed these IHC findings (Fig. 5c). Rare scattered acinar cell necrosis without ICIB was noted in 1 of 8 submandibular glands examined. Rare ductal cell necrosis in the absence of ICIB was observed in 2 of 6 sublingual glands available for review.

### Thyroid

Thyroid tissues were collected incidentally with the trachea and esophagus from 4 of 23 guinea pigs examined. In all glands, very low numbers of macrophages and fibroblasts containing ICIB were present in the interstitium surrounding the follicles. In 3 of the 4 thyroid tissues available, multifocal mild-to-moderate follicular cell degeneration and necrosis were noted with low numbers of infiltrating viable and degenerate macrophages within the follicles (Fig. 5d). Both macrophages and follicular cells contained ICIB and were immunopositive, with occasional granular staining of the colloidal material similar to the staining of plasma within blood vessels (Fig. 5e). Macrophages, interstitial fibroblasts and follicular epithelial cells were also positive for EBOV RNA by ISH (Fig. 5f).

### Adrenal Glands

**Figure 6:**
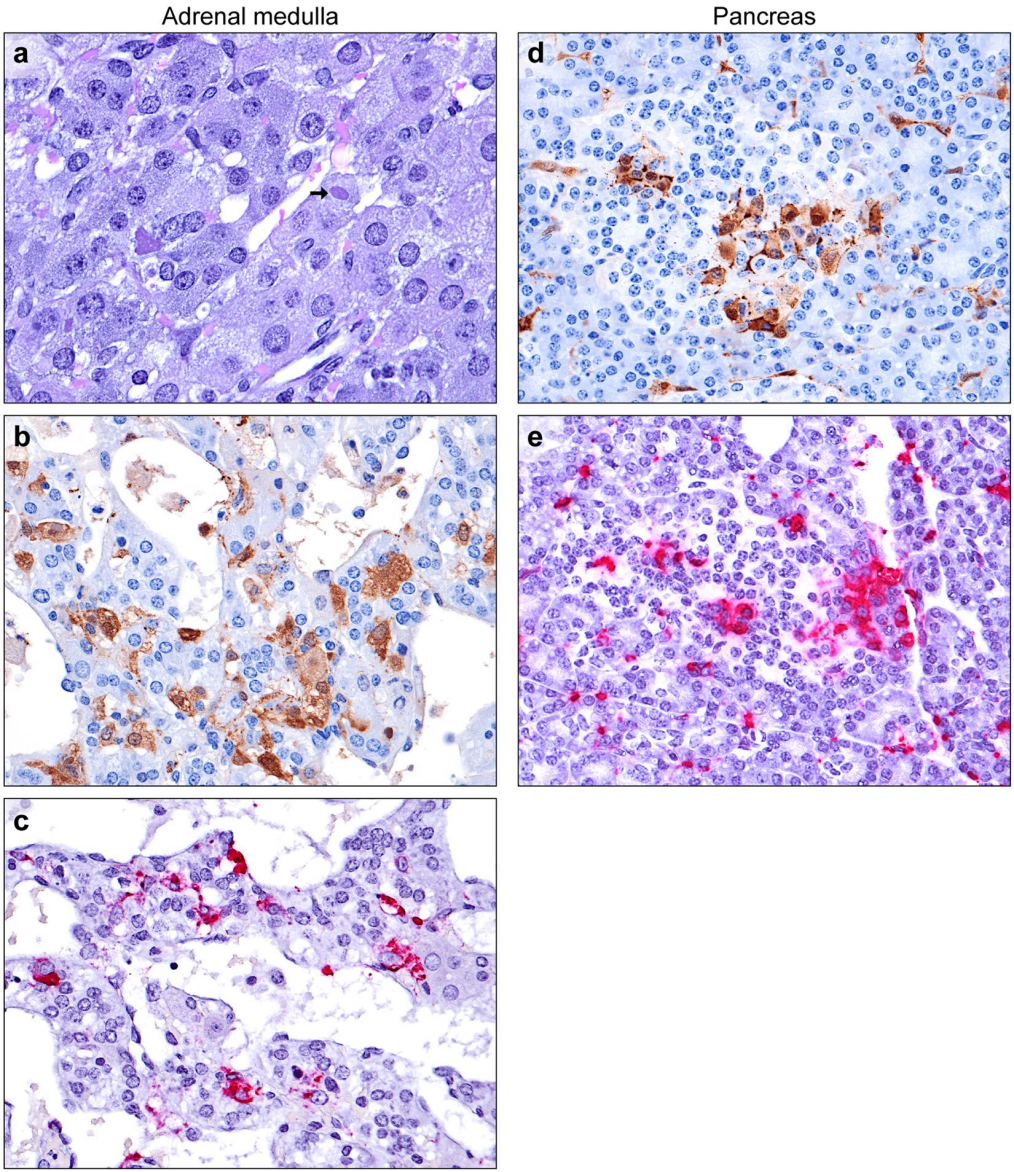
EBOV infection of adrenal medulla and pancreas. (**a**) Occasional large viral inclusions (arrows) within chromaffin cells (H&E). (**b**) Chromaffin cells (EBOV VP40 IHC: DAB chromogen and hematoxylin). (**c**) EBOV-infected chromaffin cells (EBOV NP ISH: fast red chromogen and hematoxylin). (**d** and **e**) EBOV-infected endocrine cells of the Islets of Langerhans, exocrine acinar cells, and interstitial fibroblasts and macrophages (**d**: EBOV VP40 IHC: DAB chromogen and hematoxylin, **e**: EBOV NP ISH: fast red chromogen and hematoxylin).

Consistent with previous reports on the guinea pig model, adrenal cortical cells and sinusoidal macrophages were frequently infected^30,31,33,41^. Low numbers of chromaffin cells (less than 5%) with viral ICIB were present in the adrenal medullas from 5 of the 18 guinea pigs from which they were collected (Fig. 6a–c). Glassy eosinophilic “hyaline globules” within the cytoplasm of medullary cells were noted more frequently than viral ICIB^42^.

### Pancreas

Of the 23 guinea pigs available, 6 pancreata were examined. Very low numbers of macrophages and fibroblasts containing ICIB were noted in the interstitium surrounding acini and ducts, consistent with previous reports^30^. The presence of ICIB and positive EBOV IHC and ISH signals within acinar cells were also expected. In 5 of the 6 pancreata, low numbers of endocrine cells within the islets of Langerhans contained ICIB and stained positively by IHC and ISH, frequently occurring in small clusters (Fig. 6d,e). Islets were not uniformly affected, with some islets containing no infected cells while rare islets had up to 20% EBOV infection.

## Discussion

The gross and histologic lesions of experimental EBOV-induced disease in nonhuman primate and rodent models are considered largely identical with that of natural human infections^30,31,43^. However, this presumed identity may be incorrect because, for instance, only very few autopsies of EVD victims have been performed^43^. Likewise, despite the long history of using guinea pigs in filovirus research^28–39^, many manuscripts do not include histology or are limited to the evaluation of livers and spleens. Only a handful of studies have been performed to thoroughly characterize EBOV cell tropism in the guinea pig using histology, ultrastructural evaluation and/or IHC, and almost none have utilized ISH to detect viral genome^30,31,33,41^. In our study, we discovered that EBOV infects and likely replicates in numerous tissues and cell types previously not known to be involved in the pathogenesis in the guinea pig model.

Although not previously described in filovirus infection models, the finding of oval cell hyperplasia was not unexpected because of the extensive hepatocellular necrosis subsequent to EBOV infection. Oval cells, derived from the terminal ductule epithelial cells of the canal of Hering, are bipotential progenitor cells that may differentiate to biliary cells or hepatocytes^40^. Oval cells may be induced with any liver injury, including toxins and infections. However, we were surprised by the presence of EBOV RNA and antigen in oval cells, which could potentially affect both EBOV clearance and hepatic parenchymal regeneration in EVD survivors.

EBOV infection and inflammation of the atrioventricular valves and chordae tendinae of the heart was also unexpected, but could possibly explain valvulopathy in survivors, either as a result of inflammation and scarring or of autoimmune disease. Similarly, infection of the chordae tendinae could predispose to rupture of the tendinae, resulting in atrioventricular valve prolapse. Infection of the atrial myocardium and vascular endothelium and tunica media has been previously described, but whether valves were included in previous examinations is unclear^30^. Demonstration of abundant viral antigen in the endocardium (NOS) has been reported in fatal acute infections in humans^43^.

EBOV infection of the supporting cells of the peripheral ganglia and nerves, with attendant inflammation, is intriguing, as the nature of neurologic impairment in EVD survivors is hotly debated^17,26,44,45^. Damage to these neural supporting cells, with a pro-inflammatory resolution, could conceivably lead to long-term nerve or ganglion damage, manifesting as altered sensory inputs or dysautonomia. Therefore, prospective studies of a survivor model of EVD, once established, should include thorough sampling of the peripheral nervous system to test this hypothesis.

Infection of ovarian thecal cells and stromal cells and macrophages of the ovary, oviduct, and uterine endometrium has been described previously in the guinea pig model of EVD and MVD^30,46^. Infection of smooth myocytes of the vascular tunica media has also been described^30^. Thus, the finding of smooth myocyte infection in the guinea pig uterus, although novel, was not entirely surprising. Although a minor feature, epithelial cell infection of the endometrium, vagina and penis/prepuce is highly interesting vis-à-vis potential sexual transmission of virus during acute subclinical or prodromal infection^4,19,20^. Other mucosal epithelia such as those of the tongue and esophagus have been previously demonstrated by others to be infected^30,32^.

The high incidence of EBOV infection and inflammation of the parotid salivary gland is significant, as saliva is known to contain the virus in acutely infected and recently convalescent individuals, and inflammation of the parotid gland is a documented sequela of disease in survivors^47^. The preference of EBOV for the parotid gland over the submandibular and sublingual glands may explain negative findings in a previous study, in which the specific salivary gland(s) examined was/were not reported^31^. Preferential infection of the parotid salivary gland has been described for other viruses, including a rat polyomavirus and mumps virus (MuV; *Mononegavirales*: *Paramyxoviridae*: *Mumps rubulavirus*)^48–50^.

With the exception of the adrenal cortex, infection of the endocrine system by EBOV has received little attention^30,31,33,41^. EBOV infection of the thyroid follicles and pancreatic islets has been described in rhesus monkeys with a prolonged clinical course, and previous guinea pig studies have reported virus without lesions in the thyroid interstitium^30,51^. Infection of the pancreatic islets and adrenal cortex and medulla has been reported during acute Marburg virus infection in rhesus monkeys and humans^52,53^. Virally-mediated destruction of these tissues could potentially predispose to autoimmune disease. While endocrine disorders have not been specifically reported among EVD survivors, disease presentation can be protean, and diagnosis difficult.

Investigations to see whether the infected tissues and cell types identified in our study also are infected in other animal models of EVD and whether filoviruses other than EBOV mimic the observed tropism in the various animals will be interesting. Discrepancies could possibly explain the differences between the filovirus disease animal models in terms of possible routes of infection, incubation period, presence or absence of individual clinical signs, lethality, and efficacy of candidate medical countermeasures. That said, our study has several limitations. First, we retrospectively analyzed tissues collected during a standard EBOV confirmation-of-virulence experiment. Thus, none of the examined tissues were target tissues for collection. Instead, the tissues were collected incidentally with other tissues expected to produce typical lesions of EBOV infection (e.g., liver, lymphoid organs, adrenal cortex), resulting in non-uniform sampling. A more thorough guinea pig study therefore ought to be performed with the sole focus to identify all EBOV target cells *in vivo*. Second, none of the collected tissues were analyzed by ISH for the presence of EBOV antigenomes, thereby proving EBOV replication, nor were they examined by transmission electron microscopy or for virus titers. This latter omission may have contributed to our limited ability to make correlations between infectious inoculum dose and lesion development. At this point, we therefore do not know whether the infection of the various newly identified EBOV-susceptible cell types truly leads to production of progeny virions, and consequently we cannot speculate about how much our findings truly contribute to pathogenesis. However, results of our study indicate that the pathology of filovirus infections in animal models is much less understood than previously appreciated, and that multiple opportunities may exist for filoviruses to establish cryptic and/persistent infections throughout the infected body.

## Materials and Methods

### Animals and virus

Fifteen male and 15 female Hartley guinea pigs (*Cavia porcellus*, Crl:HA, Charles River Laboratories, Wilmington, MA, strain code 051) were acclimatized to the Maximum Containment (Biosafety Level 4) Laboratory at the US National Institutes of Health (NIH)/National Institute of Allergy and Infectious Diseases (NIAID)/Division of Clinical Research (DCR)/Integrated Research Facility at Fort Detrick (IRF-Frederick). The guinea pigs were randomized to one of three dosage groups (n = 10/group) and infected by the intraperitoneal route with guinea pig-adapted Ebola virus/UTMB/C.porcellus-lab/COD/1976/Yambuku-Mayinga-GPA (BioSample ID: SAMN05755726; henceforth EBOV), receiving the targeted doses of 10, 100, or 1,000 PFU, respectively.

All work with infectious virus was conducted in a Maximum Containment (Biosafety Level 4) Laboratory at the IRF-Frederick that is fully accredited by the Association for the Assessment and Accreditation of Laboratory Animal Care International. All animal experiments were performed in accordance with animal study protocols approved by a DCR Animal Care and Use Committee. Protocols were compliant with the US Department of Agriculture Animal Welfare Act regulations and the US Public Health Service Policy on the Humane Care and Use of Laboratory Animals and adhered to the recommendations stated in The Guide for the Care and Use of Laboratory Animals.

Twenty-three of the 30 guinea pigs were humanely euthanized in accordance with defined experimental endpoints (Table 1) between d 7 and 9 post-exposure, and gross necropsy was performed by one of two American College of Veterinary Pathologists (ACVP) diplomate veterinary pathologists (SY and LH). Seven guinea pigs succumbed to disease and were not necropsied due to autolysis. Tissues were fixed for 72 h in 10% neutral buffered formalin before automated processing in a Tissue-Tek VIP-6 vacuum infiltration processor (Sakura Finetek USA, Torrance, CA) followed by paraffin embedding with a Tissue-Tek model TEC (Sakura). Slides were cut on a Leica model 2245 microtome at 4 µm, stained with hematoxylin and eosin (H&E) and coverslipped. Slides were examined by a single ACVP diplomate veterinary pathologist (TKC) blinded to intervention. All images were captured with a Leica DM3000 microscope and DFC 500 digital camera using Leica Application Suite version 4.10.0 (Leica Microsystems, Buffalo Grove, IL).

### Immunohistochemistry

EBOV immunohistochemistry (IHC) was performed with mouse anti-EBOV matrix protein (VP40) antibody (1:1500; 3G5, catalog 0201–016; IBT Bioservices, Rockville, MD) or rabbit anti-EBOV glycoprotein (GP_1,2_) antibody (1:14,000; catalog 0301-015; IBT Bioservices), followed by biotinylated anti-mouse (catalog 115-065-166, Jackson Immunoresearch Laboratories, West Grove, PA) or anti-rabbit secondary antibody (catalog 111-065-144, Jackson Immunoresearch Laboratories), and an avidin-biotin peroxidase tertiary antibody (catalog PK-6100; Vector Laboratories, Burlingame, CA). Staining was visualized with 3,3′-diaminobenzidine (DAB) chromogen (catalog BDB2004L; Biocare Medical, Concord, CA) and counterstained with hematoxylin.

### *In Situ* Hybridization

EBOV RNA *in situ* hybridization (ISH) in formalin-fixed, paraffin-embedded (FFPE) tissues was performed using the RNAscope 2.5 high definition (HD) RED kit (Advanced Cell Diagnostics, Newark, CA) according to the manufacturer’s instructions^26^. Briefly, 20 ZZ probe pairs targeting the genomic EBOV nucleoprotein (NP) gene were designed and synthesized by Advanced Cell Diagnostics (catalogue 448581). After deparaffinization with xylene, a series of ethanol washes and peroxidase blocking, sections were heated in Antigen Retrieval Buffer (Advanced Cell Diagnostics) and then digested by proteinase K (Advanced Cell Diagnostics). Sections were exposed to ISH target probe and incubated at 40 °C in a hybridization oven (HybEZ™, Advanced Cell Diagnostics) for 2 h. After rinsing, the ISH signal was amplified using company-provided Pre-amplifier and Amplifier conjugated to alkaline phosphatase (AP) and incubated with a red substrate-chromogen solution for 10 min at room temperature. Sections were then counterstained with hematoxylin, air-dried, and coverslipped.

### Data availability

Digitally scanned slides will be made available upon request.

## Electronic supplementary material

Dataset 1
