# Author Correction: Histology, immunohistochemistry, and *in situ* hybridization reveal overlooked Ebola virus target tissues in the Ebola virus disease guinea pig model

**DOI:** 10.1038/s41598-019-51386-4

**Published:** 2019-10-15

**Authors:** Timothy K. Cooper, Louis Huzella, Joshua C. Johnson, Oscar Rojas, Sri Yellayi, Mei G. Sun, Sina Bavari, Amanda Bonilla, Randy Hart, Peter B. Jahrling, Jens H. Kuhn, Xiankun Zeng

**Affiliations:** 1 Integrated Research Facility at Fort Detrick, National Institute of Allergy and Infectious Diseases, National Institutes of Health, Fort Detrick, Frederick, Maryland USA; 2 United States Army Medical Research Institute of Infectious Diseases, Fort Detrick, Frederick, Maryland USA; 3 Present Address: Path-2-Gene, LLC, Harrisburg, PA USA

Correction to: *Scientific Reports* 10.1038/s41598-018-19638-x, published online 19 January 2018

This Article contains a repeated typographical error in the legends for Figures 1, 3 and 5, where,

“amphiphilic”

should read:

“amphophilic”

