# Supplementary material for: Histology, immunohistochemistry, and in situ hybridization reveal overlooked Ebola virus target tissues in the Ebola virus disease guinea pig model
Source: Sci Rep. 2018 Jan 19;8:1250. doi: 10.1038/s41598-018-19638-x (PMC5775334; doi:10.1038/s41598-018-19638-x)
Supplement: Supplementary file 1 — Dataset 1 [file 41598_2018_19638_MOESM1_ESM.doc]

**Histology, immunohistochemistry, and in situ hybridization reveal overlooked Ebola virus target tissues in the Ebola virus disease guinea pig model**

**Timothy K. Cooper^1^*, Louis Huzella^1^, Joshua C. Johnson^1^, Oscar Rojas^1^, Sri Yellayi^1#^, Mei G. Sun^2^, Sina Bavari^2^, Amanda Bonilla^1^, Randy Hart^1^, Peter B. Jahrling^1^, Jens H. Kuhn^1^, and Xiankun Zeng^2^***

^1^Integrated Research Facility at Fort Detrick, National Institute of Allergy and Infectious Diseases, National Institutes of Health, Fort Detrick, Frederick, Maryland, USA. ^2^United States Army Medical Research Institute of Infectious Diseases, Fort Detrick, Frederick, Maryland, USA. ^#^Current affiliation: Path-2-Gene, LLC, Harrisburg, PA.

*These corresponding senior authors contributed equally to the article. T.K.C.: Integrated Research Facility at Fort Detrick (IRF-Frederick), Division of Clinical Research (DCR), National Institute of Allergy and Infectious Diseases (NIAID), National Institutes of Health (NIH), B-8200 Research Plaza, Fort Detrick, Frederick, MD 21702, USA; Phone: +1-240-236-9240; Fax: +1-301-631-7389; Email: timothy.cooper@nih.gov; X.Z. (Kevin): United States Army Medical Research Institute of Infectious Diseases (USAMRIID), 1425 Porter Street, Fort Detrick, Frederick, Maryland 21702, USA; Phone: +1-301-619-3401; Fax: +1-301-619-4627; Email: [xiankun.zeng.fn@mail.mil](mailto:xiankun.zeng.fn@mail.mil)

**Supplemental Table 1**. Pathology findings from EBOV-infected guinea pigs at necropsy

| **EBOV Dose (PFU)** | **Animal number; sex** | **Euthanasia (day)** | **Hepatic oval cells** | **Heart** | **Nerves and Ganglia** | **Genital Tract** | **Salivary gland acini**  **(type of lesion)** | **Endocrine System** |
| --- | --- | --- | --- | --- | --- | --- | --- | --- |
| 10 | 10-1; M | 9 | NP | - Tricuspid valve and mitral valves and tricuspid CT: ICIB+, IHC+ - Cardiomyocytes: ICIB+ | NE | Vas deferens: NL | NE | - Adrenal medulla: NL - Thyroid and pancreatic islets NE |
|  | 10-2; M | 8 | NE | Mitral CT: ICIB+ | - Nerves: NL - Ganglia: NE | Testis^a^ | NE | - Thyroid: NL - Adrenal medulla and pancreatic islets: NE |
|  | 10-3; M | 8 | ICIB+ | Valves or CT: NE | NE | - Testis^a^   • Seminal vesicle: NL | PSG, SMSG, SLSG: NL | NE |
|  | 10-4; F | 8 | NP | Valves or CT: NE | Nerves and ganglia: ICIB+ | Vaginal, uterine, and oviductal smooth muscle: ICIB+ | NE | - Adrenal medulla: NL - Thyroid and pancreatic islets: NE |
|  | 10-5; F | 8 | NP | Mitral and tricuspid CT: ICIB+ | - Ganglia: ICIB+, IHC+ - Nerves: ICIB+, IHC+, ISH+ | - Vaginal epithelium IHC+, ISH+ - Vaginal and uterine smooth muscle: ICIB+, IHC+, ISH+ | NE | - Pancreatic islets ICIB+, IHC+, ISH+ - Adrenal medulla NL - Thyroid NE |
|  | 10-6; F | 7 | NP | - Valves or CT: NE - Cardiomyocytes: ICIB+, IHC+ | - Nerves: ICIB+ - Ganglia: NE | Vaginal smooth muscle: ICIB+, IHC+ | NE | - Pancreatic islets: ICIB+ - Adrenal medulla: NL - Thyroid: NE |
|  | 10-7; F | 8 | NP | Mitral and tricuspid CT: ICIB+ | - Nerves: NL - Ganglia: NE | NE | - SMSG: NL - PSG and SLSG: NE | - Adrenal medulla: NL   Thyroid and pancreatic islets: NE |
| 100 | 100-1; M | 9 | NP | Mitral CT: ICIB+ | - Nerves: NL - Ganglia: NE | Testis^a^ | - PSG: ICIB+, IHC+ - SMSG, SLSG: NL | - Adrenal medulla: NL - Thyroid and pancreatic islets: NE |
|  | 100-2; M | 9 | NP | Mitral CT: NP | NE | Epididymis and vas deferens: NL | NE | - Pancreatic islets: ICIB+ - Adrenal medulla: NL - Thyroid: NE |
|  | 100-3; M | 9 | NP | Mitral and tricuspid CT: ICIB+ | NE | - Epithelium of penis and prepuce: ICIB+, IHC+ - Testis^a^ | PSG: ICIB+  SMSG: NL  SLSG: NE | - Adrenal medulla NL - Thyroid and pancreatic islets NE |
|  | 100-4; M | 9 | NP | Tricuspid valve and mitral and tricuspid CT: ICIB+, IHC+, ISH+ | Nerves and ganglia: ICIB+ | Vas deferens: NL | NE | - Pancreatic islets: ICIB+ - Adrenal medulla and thyroid: NE |
|  | 100-5; F | 7 | NP | Mitral and tricuspid CT: ICIB+ | Nerves and ganglia: NL | - Uterine smooth muscle: ICIB+, IHC+, ISH+ - Endometrial epithelial cells: IHC+ - Ovary^a^ | - PSG: ICIB+ - SMSG: NL - SLSG: NE | - Thyroid: ICIB+, IHC+, ISH+ - Adrenal medulla: NL - Pancreatic islets: NE |
|  | 100-6; F | 9 | NP | Valves or CT: NE | - Nerves: IHC+ - Ganglia: NL | NE | - PSG and SMSG: NL - SLSG: NE | - Pancreatic islets and adrenal medulla: ICIB+, IHC+ - Thyroid: NE |
|  | 100-7; F | 8 | NP | Tricuspid CT: NL | - Ganglia: ICIB+ - Nerves: NL | NE | NE | - Adrenal medulla: NL - Thyroid and pancreatic islets: NE |
|  | 100-8; F | 8 | ICIB+ | Mitral valve and CT: ICIB+ | NE | - Uterine smooth muscle: ICIB+, IHC+, ISH+ - Endometrial epithelial cells: IHC+ | - PSG: ICIB+ - SLSG: HIST (ductal necrosis) - SMSG: NE | - Thyroid: ICIB+, IHC+, ISH+ - Adrenal medulla: NL - Pancreatic islets: NE |
| 1000 | 1000-1; M | 9 | NP | Tricuspid valve and mitral and tricuspid CT: ICIB+, IHC+, ISH+ | NE | NE | - PSG: ICIB+ - SMSG: HIST (acinar necrosis) - SLSG NL | NE |
|  | 1000-2; M | 9 | ICIB+, IHC+, ISH+ | NE | - Nerves: NL - Ganglia: NE | Testis^a^ | NE | - Adrenal medulla and pancreatic islets: NL - Thyroid: NE |
|  | 1000-3; M | 7 | ICIB+, IHC+ | - Mitral and tricuspid CT: ICIB+ - Cardiomyocytes: ICIB+ | - Nerves: NL - Ganglia: NE | Testis^a^ | NE | - Adrenal medulla: ICIB+ - Pancreatic islets and thyroid: NE |
|  | 1000-4; M | 9 | NP | Mitral valve and CT: ICIB+ | Nerves: ICIB+  Ganglia: NE | Testis^a^ | NE | NE |
|  | 1000-5; F | 8 | ICIB+, IHC+, ISH+ | Mitral CT: ICIB+ | - Nerves: NL - Ganglia: NE | Vagina: NL | - PSG: ICIB+ - SLSG HIST (ductal necrosis) - SMSG: NE | - Adrenal medulla: ICIB+ - Pancreatic islets and thyroid: NE |
|  | 1000-6; F | 8 | NP | - Mitral CT: ICIB+ - Tricuspid CT: NL | - Nerves: NL - Ganglia: NE | NE | NE | - Thyroid: ICIB+, IHC+ - Adrenal medulla: NL - Pancreatic islets: NE |
|  | 1000-7; F | 9 | ICIB+, IHC+, ISH+ | Mitral CT: ICIB+ | Nerves and ganglia ICIB+, IHC+, ISH+ | - Uterine smooth muscle: ICIB+ - Ovary^a^ | - PSG: ICIB+, IHC+ - SMSG, SLSG: NE | - Adrenal medulla: ICIB+, IHC+, ISH+ - Pancreatic islets and thyroid: NE |
|  | 1000-9; F | 9 | ICIB+ | - Mitral CT: ICIB+ - Tricuspid CT: NL | NE | - Vaginal smooth muscle: ICIB+, IHC+, ISH+ - Vaginal epithelium: IHC +, ISH+ | - PSG: ICIB+, IHC+, ISH+ - SMSG, SLSG: NL | - Adrenal medulla: ICIB+ - Pancreatic islets: and thyroid: NE |

CT, chordae tendinae; ICIB, intracytoplasmic viral inclusion bodies by routine histology; HIST, routine histology; IHC, immunohistochemistry for VP40/GP_1,2_ antigens; ISH, RNAscope in situ hybridization for viral genomic NP; NE, not examined (tissue not collected); NL, no lesion (histologically normal); NP, not present; PSG, parotid salivary gland; SLSG, sublingual salivary gland; SMSG, submandibular salivary gland. ^a^ = Histologic lesions similar to previously reported findings as described in text.[1]

1. Connolly, B.M., et al.*, Pathogenesis of experimental Ebola virus infection in guinea pig*s. J Infect Dis, 1999**. 179 Suppl** 1: p. S203-17.
